# Telocytes: Active Players in the Rainbow Trout (*Oncorhynchus mykiss*) Intestinal Stem-Cell Niche

**DOI:** 10.3390/ani12010074

**Published:** 2021-12-30

**Authors:** Nicole Verdile, Rolando Pasquariello, Gloriana Cardinaletti, Emilio Tibaldi, Tiziana A. L. Brevini, Fulvio Gandolfi

**Affiliations:** 1 Department of Agricultural and Environmental Sciences, University of Milan, 20133 Milan, Italy; nicole.verdile@unimi.it (N.V.); rolando.pasquariello@unimi.it (R.P.); 2 Department of Agricultural, Food, Environmental and Animal Sciences, University of Udine, 33100 Udine, Italy; gloriana.cardinaletti@uniud.it (G.C.); emilio.tibaldi@uniud.it (E.T.); 3 Department of Health, Animal Science and Food Safety, University of Milan, 20133 Milan, Italy; tiziana.brevini@unimi.it

**Keywords:** intestine, stem-cell niche, telocytes, rainbow trout

## Abstract

**Simple Summary:**

Aquaculture is expanding due to the high demand of fish for human consumption. However, since carnivorous fish are fed using fish-derived proteins and lipids, the sustainability of this food-producing sector is debated. Therefore, feed industries and academia are dedicating their efforts to the search for suitable raw materials and more sustainable alternative feeds that are able to ensure the health of the fish. To properly evaluate the effect of these feed formulations, extensive knowledge of the intestinal morphology and physiology is required. Moreover, the intestine is a dynamic environment in which homeostasis is controlled by highly specialized intestinal stem-cell niches. These defined functional units consist of epithelial stem cells, the supportive mesenchymal cell population, and acellular components. While they have been widely studied in the mouse intestine, this understanding is almost absent in fish species. We have previously characterized the organization of the stem-cell niche in the rainbow trout intestine; here, we expand that knowledge by examining telocytes as active stromal components of the niche. Our results indicate that this cell type is also present in rainbow trout and that it represents a key mediator of intestinal homeostasis by virtue of its active interaction with the stem cells.

**Abstract:**

In order to improve the sustainability of trout farming, it is essential to develop alternatives to fish-based meals that prevent intestinal disorders and support growth performances. Therefore, an accurate knowledge of intestinal morphology and physiology is desirable. We previously described the epithelial component of the intestinal stem-cell (ISC) niche in rainbow trout *(Oncorhynchus mykiss)*, which is one of the most successfully farmed species and a representative model of the salmonids family. This work aims to expand that knowledge by investigating the niche stromal components that contribute to intestinal homeostasis. We analyzed samples belonging to five individuals collected from a local commercial farm. Histological and ultrastructural studies revealed peculiar mesenchymal cells adjacent to the epithelium that generated an intricate mesh spanning from the folds’ base to their apex. Their voluminous nuclei, limited cytoplasm and long cytoplasmic projections characterized them as telocytes (TCs). TEM analysis showed the secretion of extracellular vesicles, suggesting their functional implication in cell-to-cell communication. Furthermore, we evaluated the localization of well-defined mouse TC markers (*pdgfrα* and *foxl1*) and their relationship with the epithelial component of the niche. TCs establish a direct connection with ISCs and provide short-range signaling, which also indicates their key role as the mesenchymal component of the stem-cell niche in this species. Interestingly, the TC distribution and gene-expression pattern in rainbow trout closely overlapped with those observed in mice, indicating that they have the same functions in both species. These results substantially improve our understanding of the mechanisms regulating intestinal homeostasis and will enable a more detailed evaluation of innovative feed effects.

## 1. Introduction

Aquaculture is increasingly becoming a key food-producing sector, providing more than half of all of the fish intended for human consumption [1,2]. However, most of them are carnivorous and, consequently, are fed with a sizable aliquot of fishmeal and fish-oil that are obtained by processing small, oily fish species caught for non-food purposes. This common practice limits aquaculture sustainability, so the industry is trying to gradually substitute the marine-derived proteins and lipids with alternative feeds derived from more sustainable raw materials [3,4]. These novel feed formulations must ensure animal welfare, optimal animal immune-system status as well as digestive-tract health. Therefore, continuous and extensive raw-material-evaluation programs together with a detailed knowledge of the renewal and repair mechanisms of the gastrointestinal (GI) tract are essential to predict and assess their final effect.

The digestive system is an extremely dynamic environment. It represents the first barrier of defense against micro-organisms and xenobiotics while simultaneously acting as the major site of digestion and absorption of macromolecules and nutrients [5,6]. Its physiological health is meticulously ensured by a sophisticated and well-defined microenvironment called the intestinal stem-cell niche [7], which consists of specialized multipotent cells known as intestinal stem cells (ISCs), subepithelial cells acting as a supportive cell population, and acellular components including growth and signaling factors [8,9].

ISCs drive and support the intestinal mucosa maintenance, giving rise to all of the differentiated epithelial cells and simultaneously preserving the stem capability through their unlimited asymmetrical division [10,11,12]. However, to ensure a proper equilibrium between self-renewal and differentiation, the niche epithelial constituents actively cooperate and interact with their stromal counterpart [13,14]. 

To date, by far the most detailed knowledge on the interaction of the epithelial and stromal components of the niche has been achieved in mice and humans [15,16]. 

We recently identified and characterized in detail the organization and distribution of intestinal stem cells in the rainbow trout (RT; *Oncorhynchus mykiss*) [17], which is a member of the *Salmonidae* family, and can be successfully farmed because of its predisposition and suitability to a wide range of farming conditions [1]. 

Although the fundamental mechanisms are conserved among different species, some significant differences have been described. We recently described that while the typical mouse IESCs and regulatory-molecule markers (*Lgr5*, *Hopx*, *Sox9*, *Notch1*, *Wnt3a* and *Dll1*) are also expressed in the RT intestine, their functional role is not conserved [17]. Indeed, in RT, *sox9* is selectively expressed by stem progenitors’ cells located at the base of the folds rather than by a transient population towards differentiation. Additionally, *hopx* is typically expressed by a rare quiescent cell population that acts as a stem-cell reserve in non-physiological conditions in mice, and in RT it identifies a pool of highly proliferating cells that rapidly expand and, in turn, generates the fully differentiated cell types. Finally, *lgr5*, which is generally considered the crypt epithelial stem-cell marker, is exclusively expressed by a mesenchymal cell population in RT, indicating the existence of an interaction between stromal and epithelial cells [17].

Experimental data demonstrated that intestinal stem cells cannot proliferate without the supplementation of essential supporting signals and factors (e.g., R-spondin, Wnt3a, Noggin, epidermal growth factor) that are partially provided *in vivo* by the surrounding mesenchymal component of the niche [15]. Among others, subepithelial telocytes (TCs) have been recently identified as a key source of signaling factors without which stem cells cannot proliferate or, consequently, support intestinal homeostasis [16]. TCs are interstitial cells characterized by a small cell body and long, thin cytoplasmic projections known as telopods (TPs) [18]. They are distributed in different tissues and organs where they have a variety of functions [19]. Their functional role is not yet fully understood, but evidence strongly suggests that this cell population is involved in many key aspects of cell biology, including cell signaling, tissue repair, and immune response [18,19].

In human and mouse intestines, they create a 3D network along the crypt–villus axis, providing both structural and functional support [18,20]. Therefore, the aim of this study was to expand our knowledge of the RT intestinal stem-cell niche by focusing on its stromal component. In particular, we evaluated the presence of telocytes along the rainbow trout gut, assessing their possible interaction with the niche epithelial components and their functional implications as key mediators of homeostasis maintenance. An accurate characterization of these mesenchymal cell populations would be helpful to further elucidate and explore the molecular mechanisms involved in preserving intestinal homeostasis and consequently to develop effective and efficient alternative feedstuffs.

## 2. Materials and Methods

### 2.1. Sample Collection

Five adult female rainbow trout (RT; *Oncorhynchus mykiss*) weighing approximately 500 g were collected from fish culture ponds at the Laghi Verdi s.n.c. trout farm (Como, Italy). The sample size is comparable to previous qualitative descriptive studies performed on the same or other species [17,21,22]. Individuals involved in this study were intended for human consumption and therefore the samples were collected right after their slaughtering. 

A longitudinal incision along the ventral line was performed and the whole gastrointestinal tract was removed. Small segments of the proximal and distal intestine were collected as we previously described [23]. Samples were rapidly fixed in 10% neutral-buffered formalin for 24 h at room temperature, dehydrated in a graded series of alcohols, cleared with xylene, and embedded in paraffin. 

For transmission electron microscope (TEM) investigations, small fragments were fixed overnight with 2.5% glutaraldehyde and 4% paraformaldehyde in 0.1 M sodium cacodylate buffer at pH 7.4, washed in the same buffer, postfixed with 1% osmium tetroxide in 0.1 M sodium cacodylate buffer, rinsed several times in water and left at 4 °C overnight in a solution of 0.5% uranyl acetate. Samples were then washed and dehydrated through an ethanol series of 30, 50, 70, 80, 90, 95% in distilled water for 20 min each and finally in 100% ethanol for other 20 min. Ethanol was exchanged for an epoxy resin mixture and the samples were embedded in fresh resin at 60 °C for 48 h. Semi-thin sections were cut with an RMC Boeckeler ultramicrotome and stained with toluidine blue. Ultra-thin sections were examined using the transmission electron microscope Talos L120C (ThermoFisher, Waltham, MA, USA) operating at 120 KV. Images were acquired by a Ceta Camera 4K × 4K (ThermoFisher, Waltham, MA, USA).

### 2.2. Histology and Histochemistry

Formalin-fixed, paraffin-embedded (FFPE) samples were used to obtain thin sections of 5 µm. FFPE sections were brought to distilled water via xylene and degraded series of alcohols and subsequently stained with hematoxylin-eosin to evaluate the general morphology of the samples. Other sections were stained with specific histochemical staining to evaluate the connective tissue. These included: Periodic Acid Schiff–Alcian Blue at pH 2.5 (PAS–AB 2.5), Mallory’s triple stain, Crossman’s trichrome, and Masson’s trichrome.

### 2.3. Immunohistochemistry

Proliferative cell nuclear antigen (PCNA) was used as a marker for proliferating cells. Its specific localization and distribution were examined though indirect immunohistochemistry using the Avidin Biotin Complex method (VECTASTAIN^®^ Elite^®^ ABC, Vector Laboratories, Burlingame, CA, USA) following manufacturer instructions. Briefly, slides were brought to boiling in an antigen retrieval solution of 10 mM Tris Base, 1 mM EDTA and 0.05% Tween20 at pH 9 in a pressure cooker for 1 min. Sections were then gradually cooled at room temperature, washed in phosphate-buffered saline (PBS, pH 7.4), and immersed in a freshly prepared 3% H_2_O_2_ solution in distilled water for 15 min to quench endogenous peroxidases. Aspecific bindings were prevented by incubating sections in Normal Blocking Serum Vectastain (VECTASTAIN^®^ Elite^®^ ABC, Vector Laboratories, Burlingame, CA, USA) at room temperature for 30 min and then by incubating with anti-PCNA mouse monoclonal antibody at 1:1600 (Millipore Corporation, MAB424, Darmstadt, Germany) diluted in 4% BSA in PBS with 0.05% Tween20 for 60 min at room temperature in a humid chamber. Sections were then incubated with the adequate biotinylated secondary antibody for 30 min and then with the avidin-biotinylated horseradish peroxidase (HRP) complex (Vectastain ABC Elite KIT, Burlingame, CA, USA) for another 30 min. Sections were exposed to 3,3’-diaminobenzidine solution (ImmPACT^®^ DAB, SK-4105 Vector Laboratories, Burlingame, CA, USA), counterstained with Mayer’s hematoxylin, dehydrated and permanently mounted.

### 2.4. Target Probe Design and In Situ Hybridization

We selected platelet-derived growth factor receptor α (*Pdgfrα*) and Forkhead Box L1 (*Foxl*1) as telocyte markers since these had previously been identified in mice [20,24]. We confirmed their expression in rainbow trout intestine by PCR and sent the sequence of the amplification product (Table S1) to Advanced Cell Diagnostics (ACD) for the design and synthesis of ad hoc custom probes (*Pdgfrα*, probe: om-pdgfra-C3, cat. No. 1029301; *Foxl*1, probe: om-foxl1-C2, cat. No. 1039271-C2).

Fluorescent in situ hybridization was performed using Multiplex Fluorescent Reagent Kit V2 (RNAscope technology, Advanced Cell Diagnostics, San Francisco, CA, USA) according to the manufacturer’s instructions. In brief, 4 µm sections were heated at 60 °C for 60 min and immediately immersed in xylene to promote paraffin removal. Slides were exposed to hydrogen peroxide (Advanced Cell Diagnostics, San Francisco, CA, USA) and brought to boiling in a target-retrieval solution (Advanced Cell Diagnostics, San Francisco, CA, USA). Subsequently, sections were incubated with Protease III (Advanced Cell Diagnostics, San Francisco, CA, USA) to encourage probes to reach their specific target. Afterward, samples were incubated with diluted probes at 1:50 in diluent buffer in a HybEZ oven (Advanced Cell Diagnostics, San Francisco, CA, USA) for 2 h at 40 °C. Probes were conjugated with different channels in order to allow multiplex comparison. Signal amplification was performed by incubating sections in signal-amplification solutions 1, 2, and 3 and then developed by applying the appropriate fluorophore (OPAL 520 or OPAL 570, Akoya biosciences, Marlborough, MA, USA) diluted at 1:750 in tyramide signal-amplification (TSA) buffer to the slides. Moreover, to evaluate the relation between the stem progenitors’ cells and the mesenchymal cell population, the two selected markers were combined for *sox9* (probe: om-Loc100135781-C3, cat. No. 847751-C3). Sections were then counterstained with DAPI and mounted with ProLong™Gold Antifade Mountant (ThermoFisher Scientific, Waltham, MA, USA). A constitutive control gene (PPiB—Peptidylprolyl isomerase B, probe: om-ppib, cat. No. 540651) was used to check the mRNA quality and integrity while a probe specific to the Bacillus subtilis dihydrodipicolinate reductase (dapB) gene was incubated as a negative control. According to the ACDRNAscope^®^ indications, the signal derived from a single mRNA molecule is detected as a dot, whereas larger dots (clusters) result from many mRNA molecules. Samples were analyzed under an Eclipse E600 microscope (Nikon, Tokyo, Japan) equipped with a digital camera (Nikon, Tokyo, Japan). Images were acquired with NIS-Elements software (NIS-Elements, version 4.6; Software for imaging; Nikon, Japan, JP, 2017.)

## 3. Results

We performed histological and ultrastructural analyses that led to the identification of TCs in the rainbow trout intestinal stroma. These were further characterized by studying the expression of *pdgfrα* and *foxl1*, two well-defined markers of mouse intestinal TCs. Finally, to investigate their relationship with the niche epithelial component we evaluated their topographical localization in relation to stem cells.

### 3.1. Identification and Characterization of Telocytes in the Rainbow Trout Gut

#### 3.1.1. Histological Analysis

**Figure 1:**
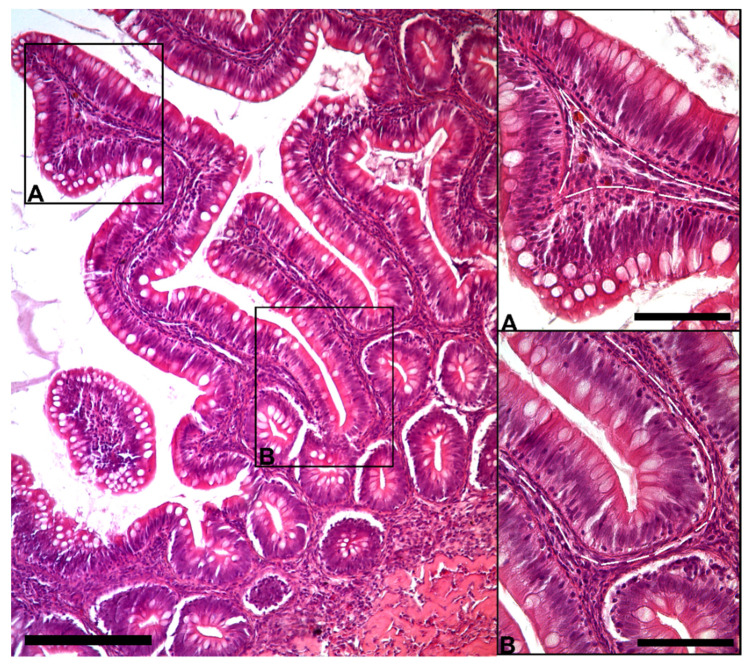
Representative image of hematoxylin–eosin-stained sections showing the general organization of peculiar stromal cells (Scale bar 250 µm) located just below the enterocytes’ basement membrane along the folds ((**A**); scale bar 50 µm) and encircling the folds’ base ((**B**); scale bar 50 µm).

The morphological analysis of the intestinal stroma revealed the presence of slender, elongated cells. They were in the subepithelial region around the folds’ base and along their length in both the proximal and distal intestine. Hematoxylin–eosin staining highlighted a typical distribution generating a supportive network within the interstitial space adjacent to the enterocytes’ basement membrane (Figure 1).

**Figure 2:**
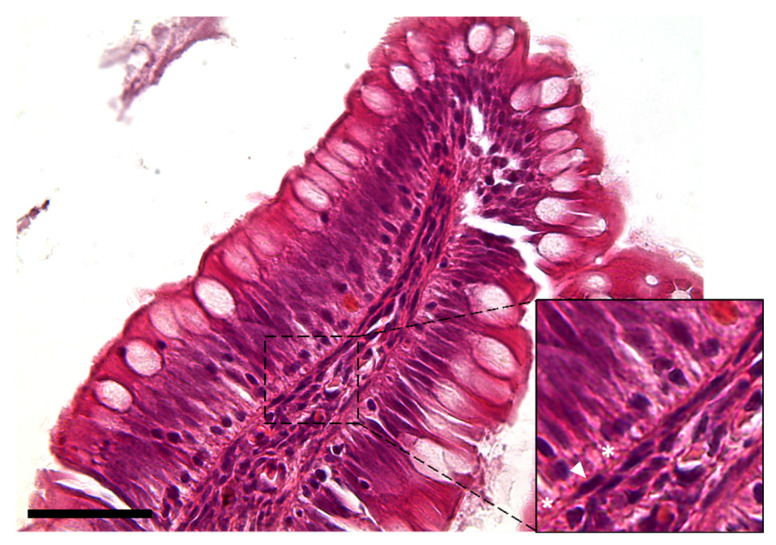
Hematoxylin–eosin-stained section showing the peculiar moniliform shape of the stromal cells because of their thin elongated nuclei (arrowhead) and long cytoplasmic branches (asterisks) (Scale bar 50 µm).

The observation using the light microscope revealed that this cell population was characterized by a peculiar moniliform shape due to the presence of a thin and elongated nucleus and stretched, slender cytoplasmic projections (Figure 2). 

**Figure 3:**
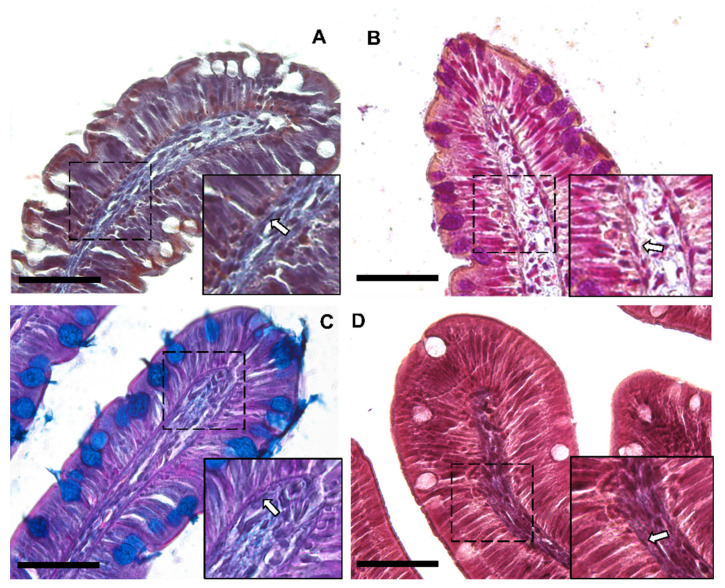
Representative images of Crossman’s trichrome (**A**), Mallory’s triple stain (**B**), Periodic Acid Schiff–Alcian Blue at pH 2.5 (PAS–AB 2.5) (**C**) and Masson’s trichrome (**D**) histochemical staining emphasizing the intricate network underneath the intestinal epithelium in the proximal intestine of rainbow trout (arrow). Scale bar 50 µm.

**Figure 4:**
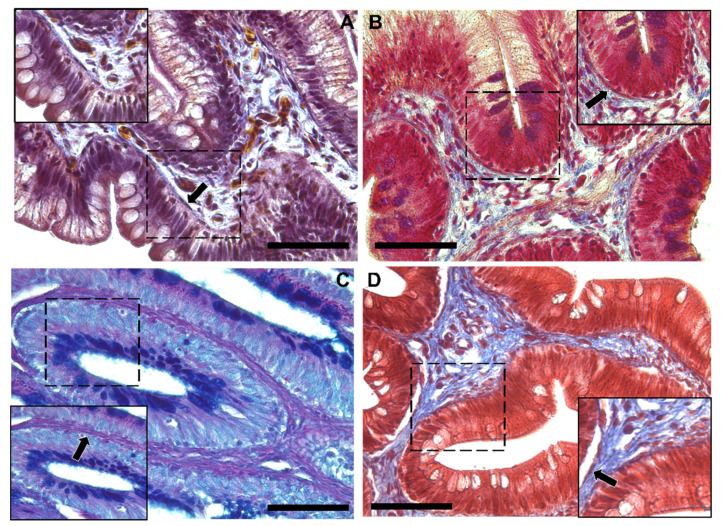
Representative images of Crossman’s trichrome (**A**), Mallory’s triple stain (**B**), Periodic Acid Schiff–Alcian Blue at pH 2.5 (PAS–AB 2.5) (**C**) and Masson’s trichrome (**D**) histochemical staining emphasizing the intricate network underneath the intestinal epithelium in the distal intestine (arrow). Scale bar 50 µm.

**Figure 5:**
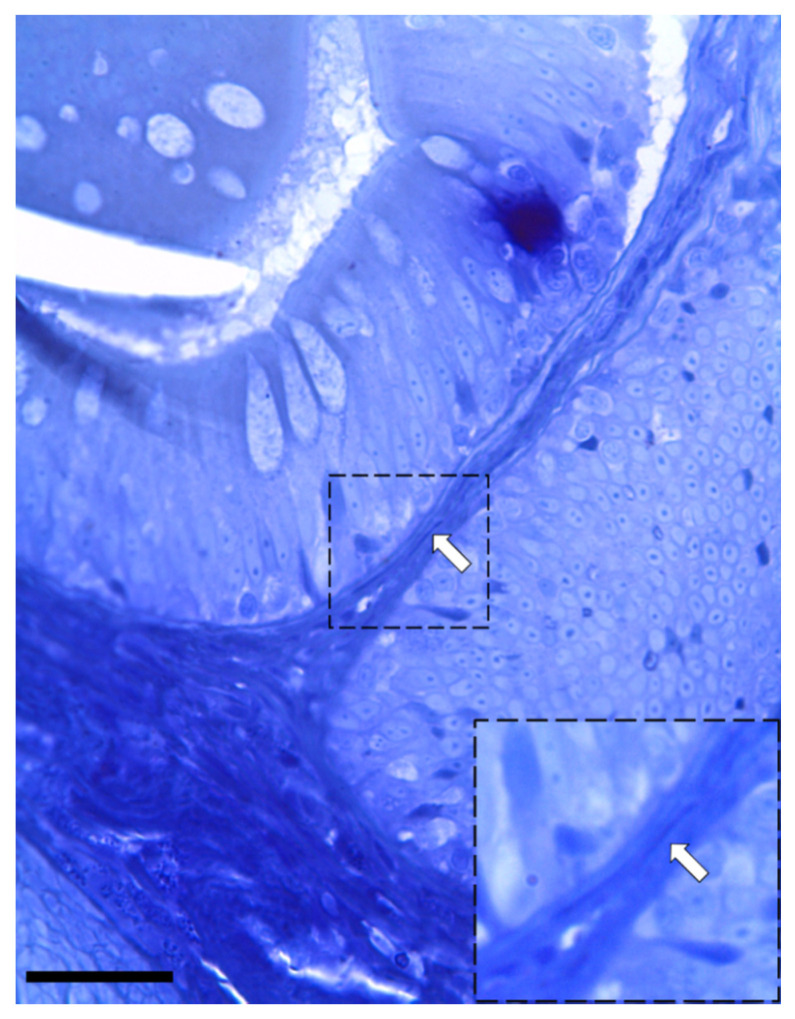
Semi-thin section stained with toluidine blue showing the presence of elongated stromal cells located along the enterocytes’ basement membrane (arrow). (Scale bar 25 µm).

Moreover, Periodic Acid Schiff–Alcian Blue at pH 2.5 (PAS–AB 2.5), Masson’s trichrome, Mallory’s triple stain, Crossman’s trichrome histochemical staining (Figure 3 and Figure 4), and toluidine-blue-stained semi-thin sections (Figure 5) further emphasized the generation of a continuous elaborate network underneath the intestinal epithelium. 

#### 3.1.2. Ultrastructural Analysis 

**Figure 6:**
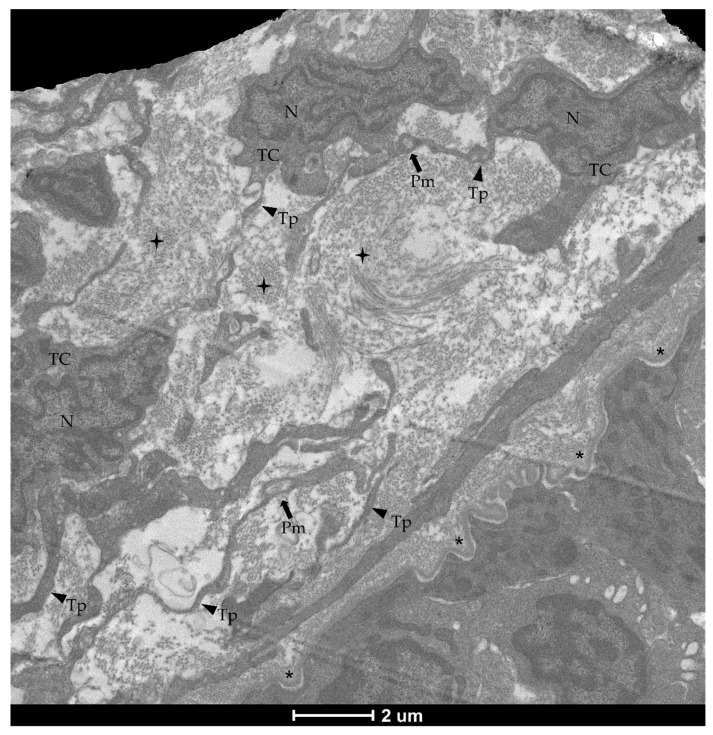
Transmission electron microscopy (TEM) showing telocytes (TCs) located along the enterocytes’ basement membrane (asterisks) characterized by extended nuclei (N), limited cytoplasm and long, thin discontinuous branches (Tp) surrounded by collagen fibers (cross) which dilate in the periphery, forming podoms (Pm).

**Figure 7:**
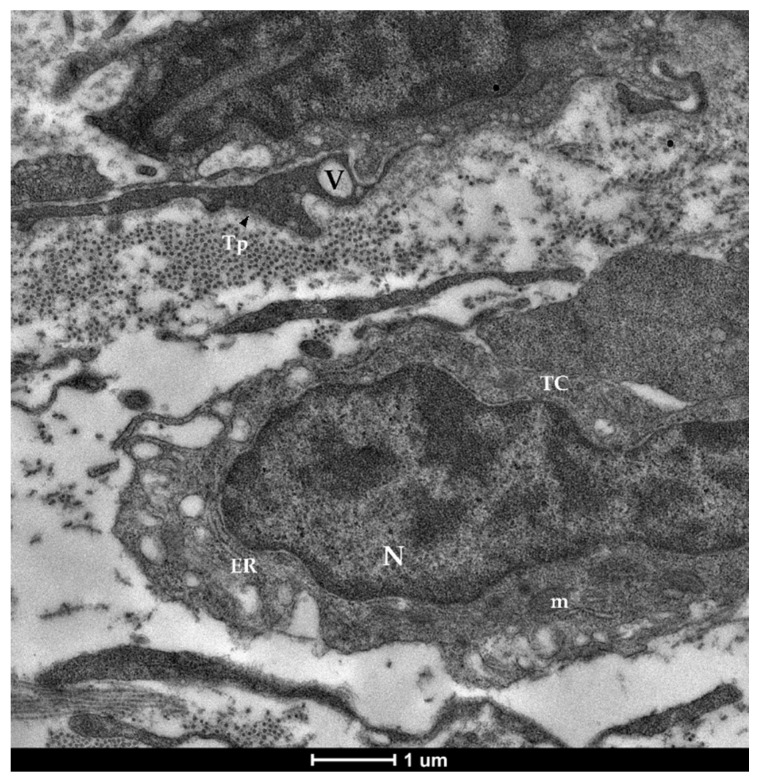
Transmission electron microscopy (TEM) showing telocytes (TCs) characterized by extended nuclei (N), limited cytoplasm hosting cellular organelles (ER—endoplasmic reticulum, m—mitochondria) and vacuoles (V).

**Figure 8:**
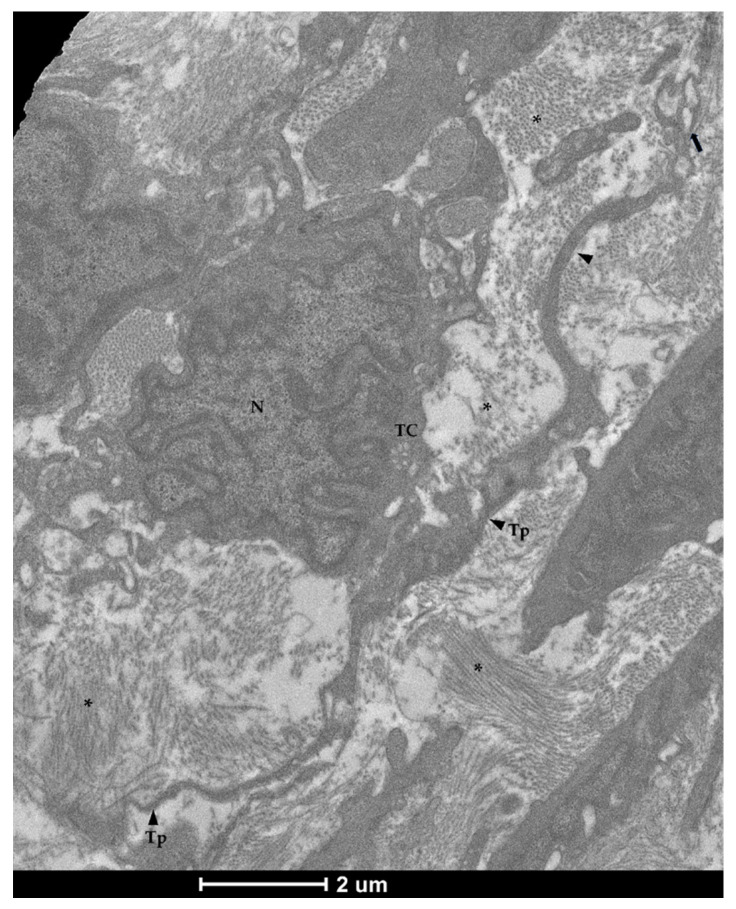
Transmission electron microscopy (TEM) showing telocytes (TCs) characterized by extended nuclei (N), scarce and limited cytoplasm with long and thin prolongations (Tp) forming podoms (arrow) and surrounding by collagen fibers (asterisks).

The examination with TEM confirmed the presence of stromal cells underlying the epithelium which were clearly distinguishable from the common fibroblasts found in the lamina propria. They presented an elongated nucleus containing heterochromatin clusters with limited cytoplasm and very long, thin projections. Moreover, they possessed an irregular shape with a variable number of branches, generally 2–3 per cell (Figure 6). These branches, called telopods (TPs), developed in a non-linear fashion, presented peripheral dilations, or podoms (Pm), that hosted cellular organelles and vacuoles (Figure 7), and were surrounded by collagen fibers (Figure 8).

Overall, histological and ultrastructural features confirmed the identity of these cells as telocytes. 

#### 3.1.3. Pdgfrα and Foxl1 Expression 

**Figure 9:**
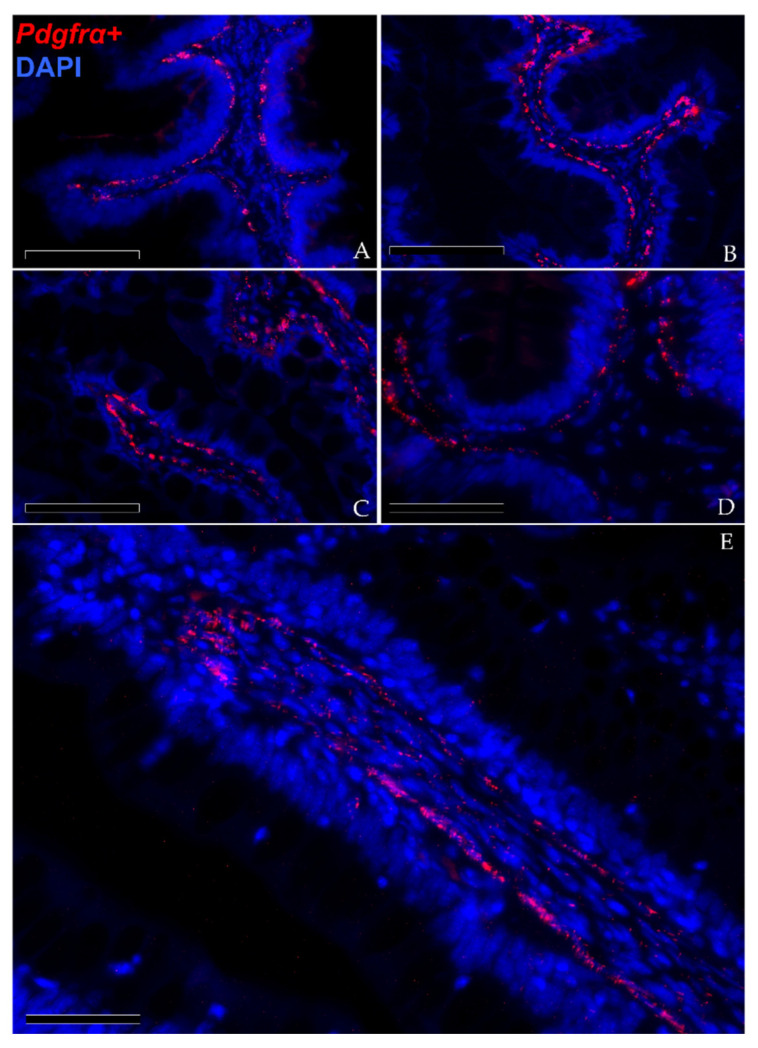
In situ hybridization of *pdgfrα* (red dots) along the rainbow trout gut. *Pdgfrα*^+^ cells were distributed along the folds’ stroma. Two distinct pdgfrα^+^ cell populations have been observed: the first showed *pdgfrα* at high levels and was specifically located adjacent to the enterocytes’ basement membrane. This created a complex mesh underlying the folds’ epithelium ((**A**,**B**); Scale bar 100 µm). Moreover, the signal was more intense along the folds’ length and at the folds’ apex compared to the one around the folds’ base ((**C**,**D**); Scale bar 50 µm). The other expressed *pdgfrα* at low levels and was located in the inner region of the lamina propria ((**E**); Scale bar 50 µm). Nuclei were counterstained with DAPI.

In situ hybridization showed that *pdgfrα* was expressed in many cells located exclusively in the stroma. Here, two clearly distinct *pdgfrα*^+^ cell populations were visible. The first displayed high *pdgfrα* expression, had slender, elongated nuclei and was located just below the basement membrane of the intestinal epithelium. These cells created a continuous plexus that extended from the basal to the apical compartment (Figure 9A,B; Scale bar 100 µm). They showed more intense *pdgfrα* expression at the fold apex and along its length, while it tended to become lower around the folds’ base (Figure 9C,D; Scale bar 50 µm). The other *pdgfrα* ^+^ population was characterized by a low signal and was located in the innermost region of the lamina propria, away from the epithelium (Figure 8E; Scale bar 50 µm).

**Figure 10:**
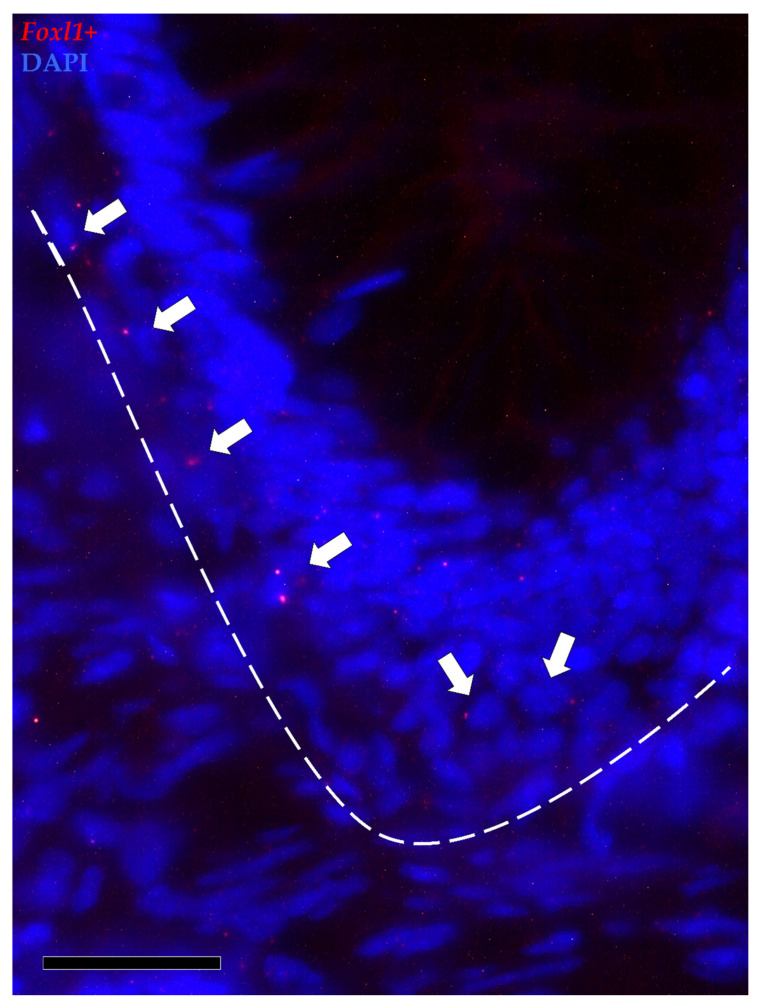
In situ hybridization of *foxl1* (red dots) around the base of the folds of the proximal intestine. *Foxl1* was expressed by a few stromal cells encircling the folds’ base (arrows). Some mRNA dots were also observed within the epithelium lining the folds’ base.

*Foxl1*+ cells were observed along the folds’ connective axis in both the proximal and distal intestine. Its expression was rare and limited to a few cells located in the peri-epithelial space at the folds’ base. In the distal intestine, *foxl1*^+^ cells were found not only around the folds’ base but also around the base of the secondary folds protruding from the complex *plicae*, which are characteristic of this region. As opposed to *pdgfr*α, scattered mRNA dots of *foxl1* were also found in the epithelium lining the base of the folds (Figure 10).

**Figure 11:**
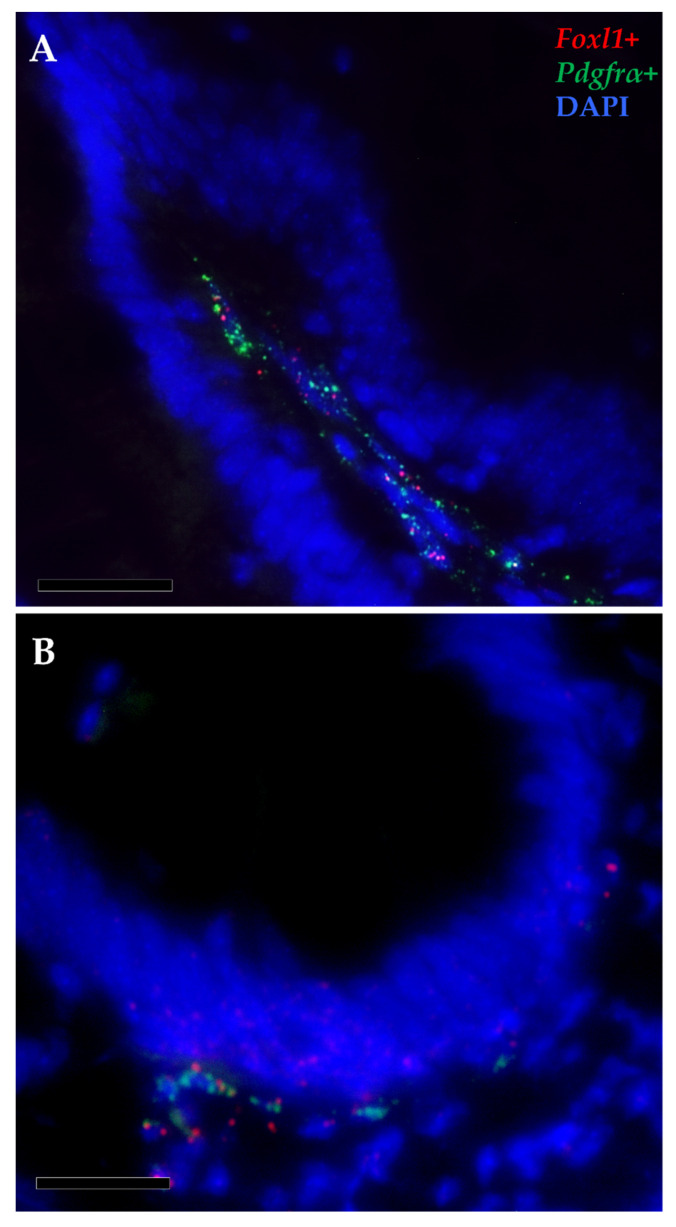
In situ hybridization of *foxl1* (red dots) and *pdgfrα* mRNA (green dots) within the stromal space along the folds (**A**) and surrounding the folds’ base of the proximal intestine (**B**). Few *pdgfrα*^+^ cells simultaneously expressed *foxl1*, indicating the presence of a small functional telocyte subset.

Furthermore, *pdgfr*α and *foxl1* transcripts were co-expressed in a few elongated cells localized just beneath the basal membrane, at the base and at the apex of the intestinal folds, indicating the presence of a small telocyte subpopulation along the whole intestinal length (Figure 11).

### 3.2. Telocytes as Stromal Component of the Stem-Cell Niche

We previously demonstrated that *sox9*+ cells in the RT intestine could represent the stem-cell population because of their morphological characteristics and topographical location being analogous to the well-known crypt-base-columnar cells (CBCs) in the small and large intestines of mice [25]. To further confirm our previous hypothesis, we performed an ultrastructural analysis of the epithelial cell population lining the folds’ base.

**Figure 12:**
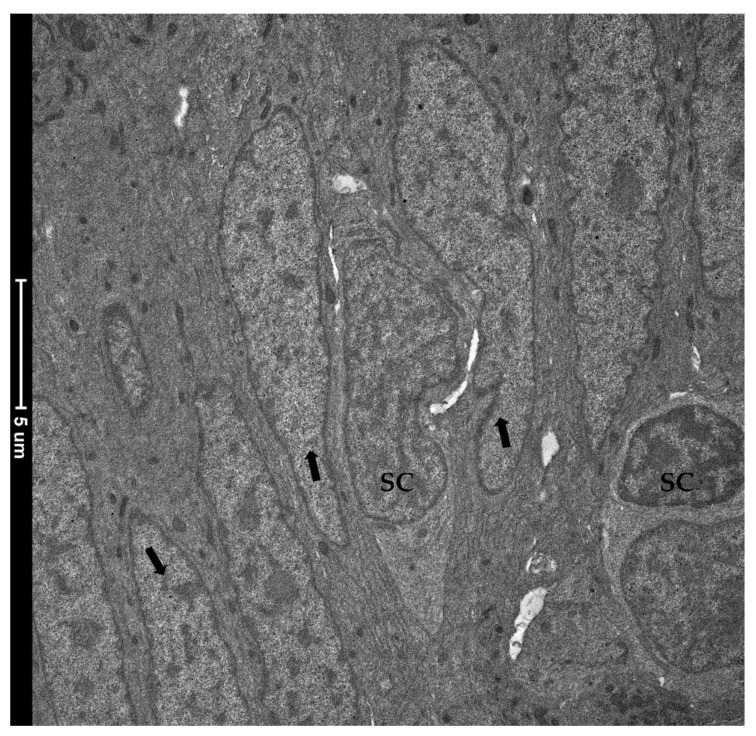
Transmission electron microscopy (TEM) showing the ultrastructural features of the epithelial cells lining the folds’ base. Rare and slender cells displaying the typical stem-cell nuclei characterized by loose, decondensed heterochromatin (SC) were found interposed among common epithelial cells defined by a heterochromatin cluster with a compact appearance (arrows).

TEM investigation revealed the presence of rare and slender cells characterized by an unbalanced nucleus-to-cytoplasm ratio in favor of the nucleus, which contained loose, decondensed heterochromatin, a distinguishing marker of the stem-cell population. These observations confirmed their identity as crypt-base-columnar stem cells. They were interposed among other common epithelial cells which were instead defined by a heterochromatin cluster with a compact appearance (Figure 12).

**Figure 13:**
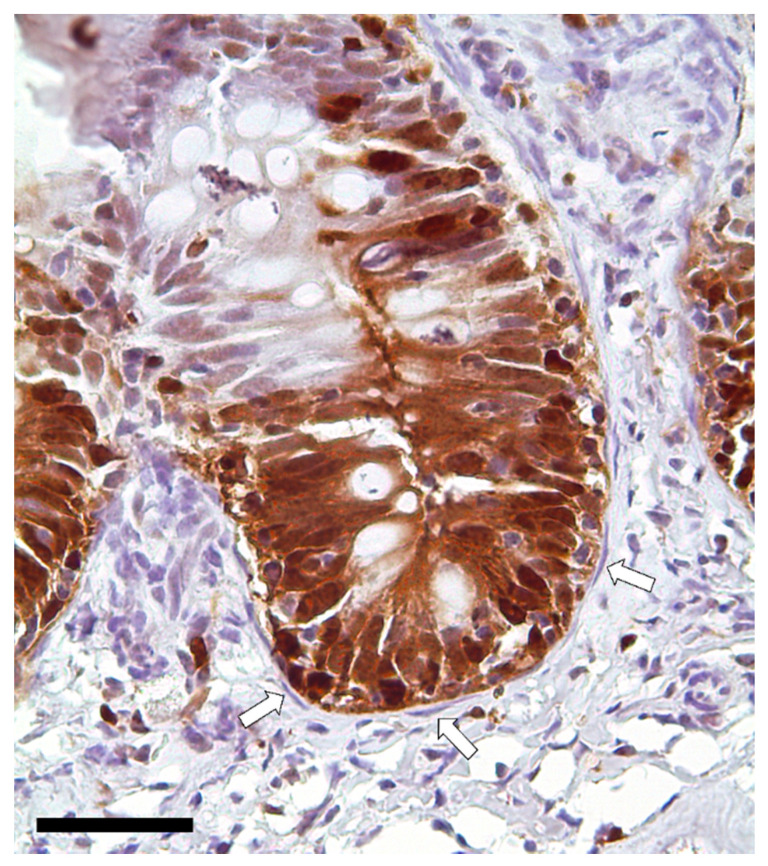
Immunolocalization of proliferating cell nuclear antigen (PCNA) showing a strong signal in the epithelial cells located at the folds’ base. Instead, telocytes enwrapping the proliferative compartment did not show any expression (arrows). Scale bar 50 µm.

Immunodetection of PCNA revealed that TCs are not proliferating cells. However, they were in direct communication with proliferating cells since they enwrapped the proliferative compartment located at the base of the folds (Figure 13). 

To further elucidate the relationship between TCs and the intestinal stem cells, we then combined the in situ hybridizations of *foxl1*^+^ and *sox9*, the marker of RT stem cells. 

**Figure 14:**
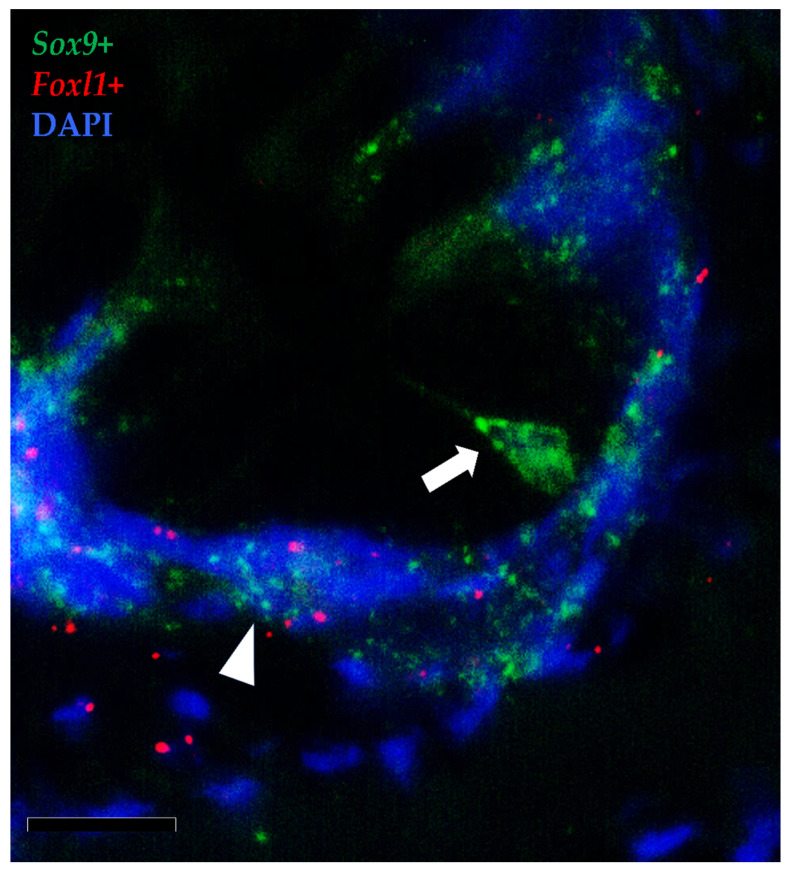
In situ hybridization of *foxl1* (red dots) and *sox9* (green dots) mRNA in rainbow trout distal intestine. Stromal *foxl1*^+^ cells were distributed in a strategic position close to crypt-base-columnar *sox9*^+^ cells (arrow). Few *foxl1*^+^ dots were also found in the epithelium lining the folds’ base where they colocalized with cells displaying *sox9* at low level (arrowhead) (Scale bar 50µm).

Moreover, the results showed that the *foxl1*^+^ TCs were in close proximity to crypt-base-columnar *sox9*^+^ cells, the typical epithelial stem cells (Figure 14). Furthermore, *foxl1* colocalized with *sox9* in a few epithelial cells expressing this gene at lower levels in the folds’ base. These are known to be cells at the beginning of their differentiation pathway, described as progenitor cells [17].

**Figure 15:**
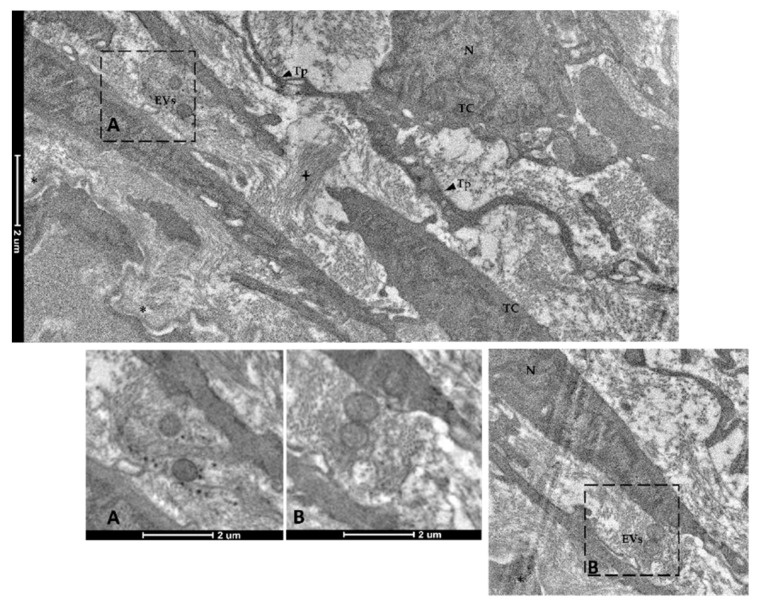
Transmission electron microscopy (TEM) showing extracellular vesicles (EVs) within the extracellular space in the close proximity of telocytes (TCs) located along the folds’ length (N—Nucleus, Tp—telopods, cross—collagen fibers, asterisk—basement membrane). Images A–B show extracellular vesicles in higher magnification.

**Figure 16:**
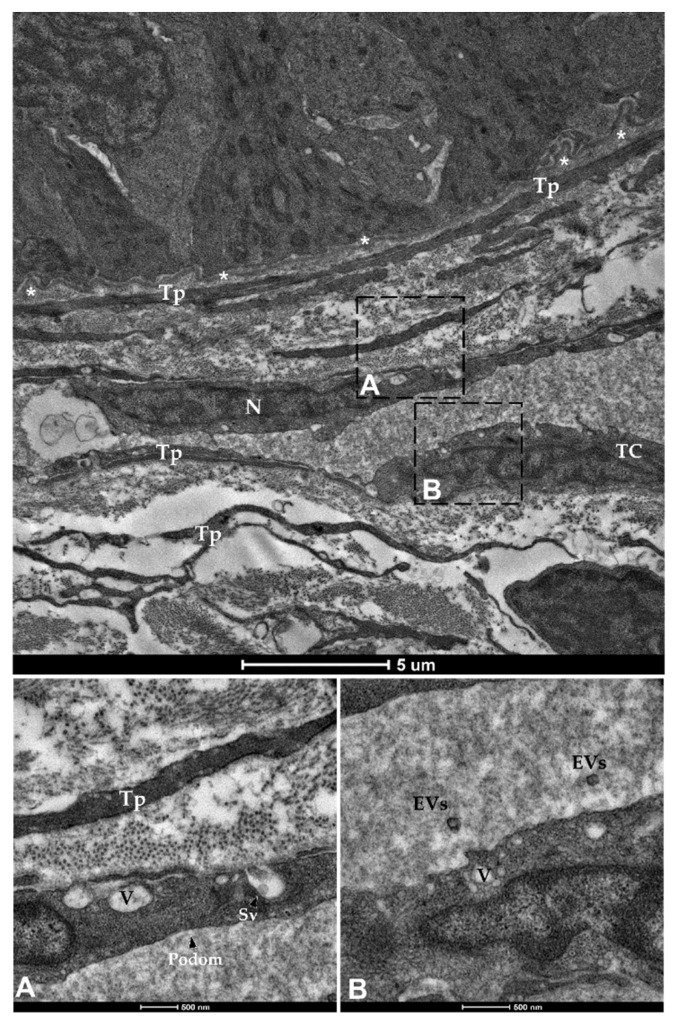
Transmission electron microscopy (TEM) showing telocytes (TCs) distributed juxtaposed to the epithelium and encircling the folds’ base, characterized by extended nuclei and long, slender prolongations. Shedding vesicles (SVs) and extracellular vesicles (EVs) released within the extracellular space are visible in higher magnification (**A**,**B**). (N—Nucleus, Tp—telopods, V—Vacuoles, asterisks—basement membrane).

Finally, spherical vesicles were detected in the extracellular space close to telocytes. Their position, shape, membrane morphology and size enabled their classification as extracellular vesicles (EVs) (Figure 15 and Figure 16). Furthermore, TCs distributed along the folds’ length produced EVs that were larger in diameter (310–450 nm) compared to those released by the TCs encircling the folds’ base (120–130 nm).

## 4. Discussion

We previously described the organization and the architecture of the rainbow trout ISCs, identifying the epithelial and stromal niche components in this species [17]. Here, we expand that knowledge focusing on the stromal population and identifying the presence of telocytes that have recently been indicated as active components of the intestinal niche.

We found peculiar stromal cells that were juxtaposed to the enterocytes’ basement membrane. They displayed the typical telocytes’ moniliform shape due to their long and thin cytoplasmic projections [16,26,27,28] and were continuously distributed underneath the epithelium extending from the folds’ base to their apex. Several histochemical stains specific to connective cells further evidenced the presence of a mesh underlying the folds’ epithelium. Its morphology, position, and distinctive organization prompted us to establish that this cell population corresponds to the telocytes widely described in mouse and human intestines [20,29]. To confirm our findings, we performed an ultrastructural analysis that is considered the gold-standard methodology for the identification of this cell type [30,31,32]. The TEM investigation revealed a typical and voluminous nucleus with scarce cytoplasm, long and discontinuous branches, and an irregular shape that varied according to the number of cytoplasmic extensions. These observations allowed us to clearly distinguish this cell population from the common fibroblasts that are located along the gut stroma. Overall, their typical ultrastructural characteristics are fully in agreement with those reported for telocytes in the intestine and in other organs of mammals and different species [26,33,34,35,36,37]. This includes the presence of extracellular vesicles (EVs) located in the interstitial space in close proximity to TCs that were classified either as microvesicles (MVs) or exosomes based on their diameter and size [38]. In humans, MVs play a key role in cellular communication by conveying proteins, messenger RNA and microRNA (miRNA) to faraway cells that modify gene expression, proliferation, and differentiation of the receiving cell [39]. Our findings support the thesis that RT TCs take advantage of these intercellular means of communication to transfer biological information and also to assure long-range cell-to-cell signaling. Moreover, while MVs were found in the close vicinity of TCs that were distributed along the folds’ length, exosomes were observed near the TCs enwrapping the folds’ base. This observation suggests that TCs exploit different forms of cell–cell communication based on their topographical localization in the stromal space.

Recently, in mice and humans, TCs have increasingly aroused interest due to their central role as mesenchymal components of the stem-cell niche. For instance, they have been described to directly interact with the stem-cell microenvironment in the heart, lungs, skeletal muscle, skin and intestine [16,20,39,40,41]. In the latter, data, almost exclusively from mice, demonstrated that intestinal telocytes provide essential signaling factors to guide and direct the fate of stem cells. In particular, they have been proposed to be a critical source of *Wnt* ligands and growth factors that actively coordinate ISC renewal and differentiation [20]. Despite the potential interest that an accurate knowledge of the mechanisms regulating the homeostasis of the intestinal wall in domestic species would have for nutrition studies, these mechanisms have been scarcely investigated. We here analyzed the expression of *pdgfr*α, which is a well-characterized marker of human and mouse mesenchymal cells in general, including telocytes [29], and is known to be crucial for the physiological gastrointestinal development and to play a key role in small intestinal mucosa morphogenesis [16,42]. We also investigated the expression of *foxl1*, another marker of intestinal TCs, that has recently been described to be a fundamental mouse-stem-cell-niche component [15,16,20]. 

In RT, *pdgfr*α^+^ cells were selectively distributed within the lamina propria all along the intestine. This observation is consistent with previous findings in mice and humans [29,43]. Stromal cells also expressed *pdgfr*α at different levels. In particular, those showing the highest expression were ascribable to TCs because they displayed elongated nuclei, were located just beneath the epithelium, and created an intricate network extending from the base of the folds towards their tips. It has been proposed that the presence of this complex mesh and the establishment of a direct connection with the overhead epithelium allows the physiological preservation of the intestinal architecture [16]. The more intense *pdgfr*α expression in the TCs located at the folds’ tip than in those encircling the folds’ base that we observed in RT has also been reported in the small intestines of mice [42,43]. In this species, single-cell RNA sequencing studies demonstrated that TCs located around the crypt base preserve the stem-cell reservoir, while those localized at the folds’ tip promote cell differentiation, thereby ensuring an efficient and effective epithelium [43]. Given the close morphological correspondence between the two species, we hypothesized that TCs also have different functions based on their topographical location in RT. 

Conversely, stromal cells displaying *pdgfr*α expression in the inner region of the lamina propria also display a rounded nucleus and lack the distinguishing features that characterize telocytes. Therefore, they should be considered as common fibroblasts of the intestinal stroma, which are not directly involved in the stem-cell-niche function. Their expression of *pdgfr*α may be attributable to other known functions of this receptor like connective-tissue remodeling [44].

In mice, *Foxl1*-expressing subepithelial telocytes are considered a critical component of the stem-cell niche [15,20]. Indeed, they provide essential *Wnt* signals to the epithelial stem and progenitor cells lining the crypt base, so much that *Foxl1* ablation results in the impairment and perturbation of the intestinal mucosa architecture followed by death within 3 days [45]. Exactly as it has been reported in mice, *foxl1*^+^ cells are also rare in RT and are preferentially distributed along the peri-epithelial space enwrapping the folds’ base [15]. Although we were not able to verify their specific functional role because genetic ablation studies are unfeasible in RT, it is reasonable to assume that RT *foxl1^+^* cells also represent an essential component of the stem-cell niche.

The dual-label fluorescence in situ hybridization of *pdgfr*α and *foxl1* demonstrated that *foxl1* is expressed only in a subset of *pdgfr*α^+^ TCs, both at the base and at the tip of the folds. The same has been observed in mice intestines where TCs express a different transcriptome according to their topographical localization within the lamina propria [24]. All of this further reveals the intricacy of the network regulating the delicate balance between proliferation and differentiation of the intestinal epithelium and highlights how these cells may represent a sensitive target for the development of functional feeds.

Immunolocalization of proliferating cell nuclear antigen (PCNA) indicated that RT TCs do not actively divide, but instead they possess a unique, well-defined phenotype, suggesting their terminally differentiated state. This is consistent with other studies in the literature in which it has been speculated that TCs may represent the progenitors’ of the mesenchymal cell population, but in pathological conditions [46]. However, TCs enwrapping the folds’ bases were located in close proximity to the epithelial proliferative compartment. This strongly suggests an interaction between the mesenchymal and the epithelial components. In addition, the in situ detection of *foxl1* combined with *sox9* demonstrated that this specific stromal population is juxtaposed to *sox9*^+^ cells. We previously demonstrated that in RT, *sox9*+ cells are the functional equivalent of the well-known, actively cycling intestinal stem cells named crypt-base-columnar cells (CBCs) in mice. Here, we further confirmed our previous findings demonstrating that these cells also display the typical decondensed heterochromatin arrangement which characterizes intestinal stem cells [47].

Therefore, it is reasonable to assume that the peri-epithelial TCs that envelope the stem-cell zone also promote their proliferation, thereby providing short-range signaling. Indeed, *foxl1*^+^ mesenchymal telocytes were also found surrounding the base of the secondary folds that branch off from the complex folds that are present in the distal intestine [23]. This supports our previous hypothesis [23] that secondary folds are maintained by a sort of local niche.

## 5. Conclusions

Our results demonstrate the presence of telocytes in the rainbow trout gut that form an intricate network spanning from the basal to the apical portion of the intestinal folds. Their morphology, topographical location and gene-expression pattern are almost identical to those observed in mice; therefore, we infer that telocytes also stimulate cell proliferation or cell differentiation depending on their topographical location in RT. 

The characterization of this mesenchymal cell population substantially improves our understanding of the renewal mechanism at play in the intestine and highlights the intricate signaling networks that are used for the maintenance of its homeostasis. This knowledge provides effective tools for a more accurate analysis of the effects of newly formulated functional feeds.

## Data Availability

Not applicable in this case.

## Supplementary Materials

The following are available online at https://www.mdpi.com/article/10.3390/ani12010074/s1, Table S1: List of primer sequences used for validating the expression of the genes analyzed by Fluorescence In Situ Hybridization whose probes were designed by Advanced Cell Diagnostics (ACD). Gene ID, amplicon size in base pairs (bp), accession number ID, forward and reverse primer sequences are reported for each gene.
