# Supplementary material for: Telocytes: Active Players in the Rainbow Trout (Oncorhynchus mykiss) Intestinal Stem-Cell Niche
Source: Animals (Basel). 2021 Dec 30;12(1):74. doi: 10.3390/ani12010074 (PMC8744786; doi:10.3390/ani12010074)
Supplement: Supplementary file 1 [file animals-12-00074-s001.zip › animals-1487767-SI.pdf]

Table S1. List of primer sequences used for validating the expression of the genes analyzed by Fluorescence *In Situ* Hybridization whose probes were designed by Advanced Cell Diagnostics (ACD). Gene ID, amplicon size in base pairs (bp), accession number ID, forward and reverse primer sequences are reported for each gene.

Gene ID (amplicon size in base pairs (bp))	Accession Number ID	Forward (F) and reverse (R) Primer Sequence (5->3)	Annealing Temperature (°C)	Reference
sox9 (366bp)	NM_001124179.1	F:TGCAGGAGTGCATCTTTGTC<br>R:GGTCAGCCTTCTTGAACTCG	60	[17]
foxl1 (214bp)	XM_021569469.2	F:GAGGAGAGGAACTCACAATCAC<br>R:AGTTTAGCAGAGCCACAGTATC	60	Own design
pdgfrα (237bp)	XM_021599407.2	F:TCCGTGACCATGGATGAAATG<br>R: GAAGAGCGTGGCAGTGTAATA	60	Own design
